# Ascertaining the Value of Noninvasive Measures Obtained Using Color Duplex Ultrasound and Central Aortic Pressure Monitoring During the Management of Cerebral Arteriovenous Malformation Resection: Protocol for a Prospective, Case Control Pilot Study

**DOI:** 10.2196/resprot.7991

**Published:** 2017-08-31

**Authors:** Kathryn J Busch, Hosen Kiat

**Affiliations:** ^1^ Faculty of Medicine and Health Sciences Macquarie University Sydney Australia; ^2^ Faculty of Medicine University of New South Wales Sydney Australia; ^3^ Faculty of Medicine Western Sydney University Sydney Australia

**Keywords:** brain, transcranial, arteriovenous malformation, color duplex, hemodynamics, pressure

## Abstract

**Background:**

Dramatic hemodynamic changes occur upon removal of an arteriovenous malformation of the brain (bAVM) with a number of potentially serious perioperative complications, such as intracranial hemorrhage and venous occlusive hypertensive syndrome. As these complications largely occur in the postoperative inpatient period, a rapid, repeatable noninvasive investigation to serially monitor relevant intracranial hemodynamics may be of benefit. Though, transcranial Doppler (TCD) and transcranial color duplex (TCCD) are techniques used and available to provide hemodynamic measurements postoperatively, the time course of hemodynamic sequences following bAVM resection remains uncertain.

**Objective:**

This is a prospective, case control pilot study conducted in participants having elective bAVM resection surgery.

**Methods:**

Each participant will undergo a preoperative color duplex ultrasound (CDU) of the bilateral extracranial carotid arteries, a CDU of the circle of Willis including the bAVM vessels, and a central aortic pressure measurement, repeated daily, postoperatively, for a 2-week period.

**Results:**

Patient accrual has commenced with anticipation of first results in 2018.

**Conclusions:**

This protocol aims to strengthen the work of previous authors by providing documentation of the time course of hemodynamic changes following bAVM resection. The protocol is designed to determine whether noninvasive technology, including CDU imaging of the extracranial carotid and intracranial arteries in the form of TCCD along with central aortic pressure measurements, can determine whether there are any hemodynamically significant prognostic markers that may provide insight into the process of vessel remodeling, including insight into venous changes following bAVM resection.

## Introduction

Dramatic hemodynamic changes occur upon removal of an arteriovenous malformation of the brain (bAVM) [1-9]. Before the vasculature returns to normal, the velocity in the former large arteries and veins is markedly reduced concurrent with an elevation of arterial pressure, which is associated with a number of potentially serious postoperative complications, such as arterial-capillary-venous hypertensive (ACVH) syndromes including hemorrhage, edema and vasospasm [10]. In our institution, by rigorously controlling blood pressure, postoperative hemorrhage has reduced from 4.4% to 1% [11].

Accordingly, as these complications largely occur in the postoperative inpatient period, a rapid, repeatable noninvasive investigation to serially monitor relevant intracranial hemodynamics may be of clinical and prognostic benefit.

Transcranial Doppler (TCD) and transcranial color duplex (TCCD) studies of cerebral arteriovenous malformations (bAVM) have established that the feeding arteries exhibit a high-velocity and low-pulsatility flow preoperatively, followed by a postoperative decrease in velocity and increase in pulsatility of the feeding arteries [2,12-21]. A recent systematic review indicated that the time course of hemodynamic sequences remains uncertain; the existing conclusions were of limited clinical value due to variations in pathology, methodology, and timing of measurements during the postoperative period, and that few studies (n=2) have used CDU applied transcranially (TCCD) to evaluate postoperative hemodynamic changes. The review also identified that the venous aspect of bAVMs using transcranial ultrasound remains largely unchartered, possibly due to technical limitations, and, to our knowledge, no study to date has used TCCD to assess both the venous components of a bAVM postoperatively using TCCD.

Proponents of TCCD suggest that in contrast to TCD, TCCD purportedly offers a higher degree of accuracy and reproducibility of velocity measurements enhanced by the ability to visually control sample volume placement [22,23], which can be problematic on TCD given that vessels change waveform characteristics following bAVM resection, making repeated measurements difficult and unreliable. Qualitative advantages of TCCD also include enhanced differentiation between feeding arteries and draining veins in patients with bAVM [23,24].

Further insight into the complex hemodynamics involved in bAVM patients is desirable, especially if it can be derived noninvasively.

## Methods

### Study Design

This is a prospective, case control pilot study conducted in participants having elective bAVM resection surgery. Each participant will undergo a preoperative CDU of the bilateral extracranial carotid arteries, a TCCD of the circle of Willis, including the bAVM vessels and a concurrent central aortic pressure measurement. This examination will be repeated daily for a 2- week period postoperatively or as long as the patient remains hospitalized between 2 PM and 4 PM to reduce diurnal variations. This protocol sequence will be referred to as the “noninvasive protocol.”

The primary outcome measure is to use TCCD and central aortic pressure measurements to establish the time course of hemodynamic changes following bAVM resection, and to determine whether there are any hemodynamically significant prognostic markers that may provide insight into the process of vessel remodeling.

The secondary outcome measure is to determine whether TCCD can provide insight into venous changes following bAVM resection.

Macquarie University Human Research Ethics Committee approved this study (Medical Sciences; reference: 5201400098).

### Participants

Eligibility criteria includes adults (>18 years) with willingness and cognitive ability to provide written and informed consent: a control group of healthy participants, a cohort of participants undergoing elective surgical resection with bAVM, and a cohort of participants undergoing elective craniotomies for tumor resection or cerebral aneurysm surgery.

Participants with a history of a psychological illness or other conditions, which may interfere with their ability to understand the study requirements, will be excluded. Participants expressing anxiety or unwillingness following initial consent will be further excluded. For the healthy volunteer cohort, pregnant women and individuals taking medication or hormonal treatment will be excluded.

### Control Group

Participants (n=20) will undergo a CDU assessment of the extracranial carotid arteries and TCCD of the circle of Willis in accordance to the protocol. The participants will be comprised of healthy volunteers recruited from research, hospital, and clinic staff at Macquarie University Hospital, and relatives. The examination will be conducted once by a dedicated vascular research sonographer (KB), and then repeated 24 hours later under the same conditions for the purpose of calculating intraobserver measurement reproducibility. The control group will be selected to represent a distribution of ages and sexes.

### Arteriovenous Malformation of the Brain Group

The bAVM group includes participants undergoing elective surgical resection of a bAVM. Participants will be recruited from the Department of Neurosurgery, Faculty of Medicine and Health Sciences, Macquarie University. Participants will be enrolled into the study following computed tomography confirmation of an operable bAVM. After the informed consent process has been completed, study participants will receive a study enrollment number and this will be recorded on all study documents.

The participants will undergo the noninvasive protocol by the research sonographer (KB). There will be no change in the participants’ routine pre- or postoperative care. Routine digital subtraction angiography will be used to correlate TCCD findings.

### Non–Arteriovenous Malformation of the Brain Group

The non-bAVM group includes participants undergoing elective intracranial aneurysm or tumor surgery. Participants will be recruited from the Department of Neurosurgery, Faculty of Medicine and Health Sciences, Macquarie University. Participants will be recruited to establish whether there exists a significant variance in hemodynamic measurements between the bAVM and non-bAVM group. By contrasting the 2 groups, it may assist in identifying hemodynamic changes of vessel remodeling in bAVM participants as the hemodynamic changes in the non-bAVM group are anticipated to be less dramatic.

The participants will undergo the noninvasive protocol by the vascular research sonographer (KB). Although the noninvasive protocol will be adhered to, data measurements obtained in this group may be for a shorter duration postoperatively, according to varied patient recovery and earlier hospital discharge rates.

### Imaging Protocol

The control and study participants will be positioned supine, with the head elevated to approximately 30°, typifying the position of postoperative bAVM patients in the intensive care unit.

The imaging component of the noninvasive protocol includes: (1) full CDU assessment of the extracranial carotid and vertebral arteries, and (2) TCCD assessment of the circle of Willis and cerebral veins, where possible.

### Carotid and Vertebral Color Duplex Ultrasound Measurements

The core vascular research sonographer (KB) at Macquarie University Hospital will perform CDU measurements of the carotid and vertebral arteries using a Philips IU22 system and a 9-2 MHz linear transducer. Peak systolic and end diastolic velocity measurements of the distal common carotid, proximal external carotid artery, proximal and distal extracranial internal carotid, and the vertebral artery will be recorded.

In the presence of atherosclerotic disease, maximal peak systolic and end diastolic velocities will be recorded at appropriate locations with stenoses graded accordingly to published diagnostic criteria.

In the absence of carotid disease, peak systolic velocity measurements will be obtained at reproducible locations by convention. Velocity measurements will be obtained by placing the sample volume cursor center stream, parallel to the lumen, and using an angle of 60° for reproducibility of measurements.

### Transcranial Color Duplex Measurements

The vascular research sonographer unit at Macquarie University Hospital will perform TCCD measurements using a Philips IU22, 5-1 MHz phased array transducer.

Although the American Society of Neuroimaging Practice Guidelines recommendation advocates assessing the entire circle of Willis, our protocol will assess the middle cerebral artery, anterior cerebral artery, internal carotid artery, and posterior cerebral artery using a transtemporal approach bilaterally. The site of maximal velocity will be recorded. The imaging protocol is based on our experience, whereby following bAVM surgery the evaluation of the entire circle of Willis using windows other than the transtemporal approach is impractical due to lowered patient tolerance levels and imposed restrictions of patient head movement in the intensive care unit.

Attempts to measure cerebral veins will be recorded and repeated using the visual reproducibility capability of TCCD. Normal reference values will be obtained by published criteria obtained from the literature and our control group.

### Noninvasive Central Aortic Blood Pressure Measurements

The Uscom BP PLUS device derives central pressure measurements that correlate closely with those obtained invasively from a catheter.

This noninvasive blood pressure device estimates the central pressures from brachial cuff pressure fluctuations, using oscillometry to determine brachial systolic and diastolic pressures during deflation of the cuff. A second inflation, holds cuff pressure approximately 30 mm Hg above the brachial systolic pressure (ie, suprasystolic measurement) for approximately 10 seconds. The intra-arterial pressures in the brachial artery at the cuff measurement site then estimate the aortic pressure using a physics-based model of the left subclavian-to-brachial branch.

This device will be used for the noninvasive protocol at the conclusion of the imaging studies, to obtain a noninvasive pressure measurement otherwise derived from invasive catheterization.

### Statistical Analysis

#### Inter- and Intraobserver Reliability

A Kappa weighted test will be performed on the 20 control participants to determine intraobserver agreement of measurements by comparing CDU of the carotid arteries and TCCD measurements of the circle of Willis 24 hours apart under the same conditions by the core vascular research sonographer. Intraoberserver reliability will be calculated.

#### Arteriovenous Malformation of the Brain and Non–Arteriovenous Malformation of the Brain Groups

Daily velocity and pulsatility measurements will be entered for each sampled vessel and plotted onto an excel spreadsheet against simultaneous daily pressure measurements. Appropriate statistical analysis will be performed according to perceived trends.

## Results

This pilot study is in progress. The data collection has commenced and the findings are planned to be completed in 2018. The results will be submitted to a leading journal for publication.

## Discussion

### Rationale for Daily Noninvasive Monitoring Following Arteriovenous Malformation Resection

Given the intensive hemodynamic changes that occur following resection or embolization of a bAVM, there is a requirement for stringent blood pressure monitoring and an extended stay in the intensive care unit given the propensity for ACVH complications including edema and hemorrhage [10,11]. Despite progressive advancement in diagnostic armamentarium for hemodynamic measurements, the precise mechanisms for postoperative bAVM hemorrhagic complications remain controversial [2-9,11]. In 1980, prior to TCD and TCCD, using pulsed echo Doppler flowmeters, Nornes and Grip commented:

Blood flow in veins has not received the attention it deserves in relation to disease in man. The dynamics of a bAVM involve venous factors of considerable significance. A bAVM hemorrhage is in principle a venous bleed caused by the increased pressure transmitted through the shunt. The arterial pulses are mediated through the shunt to the venous side. The mean velocity is at the level normally found in cerebral arteries during operations for other diseases, and considerably above what we consider normal intravenous velocities (unpublished data).

25

Since 1986, numerous TCD and later (1990) TCCD have studied bAVM hemodynamics post intervention [2,12-17,19-21,26], yet the temporal hemodynamic course following bAVM resection remains uncertain. This is due to variations in pathology, methodology, and timing of measurements during the postoperative period. Furthermore, the venous aspect following bAVM resection using either of these techniques remains unchartered [9].

This is therefore, a field of research we believe is appropriate to pursue.

### Limitations of Transcranial Doppler and Transcranial Color Duplex

Despite our aspirations in gaining insight into the venous aspect of bAVMs post resection, there are technical limitations that may prevent successful TCCD imaging of the venous aspect of bAVMs. These include difficulty delineating the boundaries of an artery and vein due to arterialization of the venous signal [24]. Another factor perhaps influencing the omission of venous flow characteristics from previous studies may relate to the lower velocity spectrum, and the close proximity to the arteries that display a more prominent signal [27]. Regardless of such mitigating factors, it remains a worthwhile consideration to attempt to include whatever hemodynamic data can be gleaned from the venous aspect of bAVMs, particularly in the postoperative phase.

Aside from the potential limitations of obtaining meaningful venous data, there may be further likely constraints regarding the feasibility of gaining useful hemodynamic information using TCCD, particularly if there is a poor temporal window or if the bAVMs are small or distally located. Therefore, this study has been implemented initially as a pilot study to assess the viability of obtaining temporal arterial and venous hemodynamic data on a range of bAVM sizes of varied locations. It may be useful, though it also may be technically difficult or even impossible, to perform evaluation of the distal vessels to the AVM and study the changes of autoregulation through the pulsatility index/hyperemic response after AVM resections. Publication of the data obtained from this pilot study will therefore include any such technical limitations.

### Conclusion

Both TCCD and CDU are noninvasive modalities that may potentially address some of the gaps in knowledge with regard to the hemodynamic remodeling following the removal of a bAVM from the cerebral vasculature, by providing simultaneous velocity and pulsatility measurements.

This protocol aims to strengthen the work of previous authors by providing documentation of the time course of hemodynamic changes following bAVM resection. The protocol is designed to determine whether noninvasive technology including CDU imaging of the extracranial carotid and intracranial arteries in the form of TCCD, along with central aortic pressure measurements can determine whether there are any hemodynamically significant prognostic markers that may provide insight into the process of vessel remodeling, including insight into venous changes following bAVM resection.

## Abbreviations

ACVH: arterial-capillary-venous hypertensive
bAVM: arteriovenous malformation of the brain
CDU: color duplex ultrasound
TCD: transcranial Doppler
TCCD: transcranial color duplex
